# Cytomegalovirus retinitis (CMVR) in an HIV-negative patient with a history of chronic lymphoproliferative disease

**DOI:** 10.1186/s12348-025-00545-y

**Published:** 2025-11-17

**Authors:** Marko Lukic, Ian Yeung, Li Qing, Mark Westcott

**Affiliations:** 1 NIHR Biomedical Research Centre at Moorfields Eye Hospital NHS Foundation Trust and UCL Institute of Ophthalmology, 162 City Road, London, EC1V 2PD UK; 2 Department of Ophthalmology, LKS Faculty of Medicine, The University of Hong Kong, Hong Kong, China; 3 William Harvey Institute Queen Mary University, London, UK; 4 Medical Retina & Uveitis Service, Moorfields Eye Hospital, 162 City Road, London, EC1V 2PD UK

**Keywords:** CMV retinitis, Macular oedema, Occlusive retinopathy, Optical coherence tomography, Immune recovery uveitis

## Abstract

**Purpose:**

To report a challenging case of cytomegalovirus retinitis (CMVR) in a Human immunodeficiency virus (HIV) negative patient with diabetes mellitus.

**Methods:**

This is a case report about a patient who was diagnosed with diabetes mellitus type 2 and treated for chronic lymphoproliferative disease (CLD). The patient developed clinical signs of cytomegalovirus retinitis (CMVR). The patient underwent a thorough diagnostic workup and complex treatment. We present the outcomes of treatment in this challenging case.

**Results:**

67-year-old man initially diagnosed with both eyes mild non-proliferative diabetic retinopathy (NPDR) presented with blurred vision in the right eye. The patient presented with hypertensive anterior CMV-related anterior uveitis. The baseline visual acuities were 6/9 in both eyes with elevated IOP in the right eye (31mmHg). Dilated fundus examination revealed right eye peripheral retinitis accompanied by haemorrhages. The anterior tap confirmed the presence of CMV, and antiviral treatment has been commenced. Despite the treatment with antiviral agents, the patient developed a right eye occlusive disease along with macular edema. The patient underwent right eye pan-retinal photocoagulation (PRP) laser therapy to address occlusive and non-perfused areas in conjunction with intravitreal anti-VEGF agents. Over time, the patient achieved disease quiescence.

**Conclusion:**

CMV retinitis in immunocompromised, HIV-negative patients with diabetes presents significant diagnostic and therapeutic challenges. Importantly, the potential development of immune recovery uveitis (IRU) – like picture should be carefully considered during the recovery phase, as it may contribute to further vision loss if not promptly recognized and managed. This case highlights the importance of individualized, multidisciplinary care and close monitoring during both the active and post-treatment phases.

## Introduction

Cytomegalovirus retinitis (CMVR) is a sight-threatening opportunistic retinal infection most classically associated with acquired immunodeficiency syndrome (AIDS) patients, where its prevalence before the era of highly active antiretroviral therapy (HAART) exceeded 30% [1]. The introduction of HAART has dramatically reduced the incidence of CMVR in people living with HIV. However, CMVR continues to pose a clinical challenge in other immunocompromised populations, including organ transplant recipients, individuals receiving systemic immunosuppressants, and patients with hematologic malignancies or metabolic diseases such as diabetes mellitus (DM) [2, 3]. Increasing reports over the last decade highlight CMVR occurring in HIV-negative patients, often with delayed diagnosis and atypical presentations [4, 5]. 

Among these non-HIV populations, diabetes mellitus and hematologic malignancies, especially leukemias, have emerged as significant risk factors for CMVR. Hyperglycemia impairs both innate and adaptive immunity, and diabetic patients are susceptible to CMV reactivation even in the absence of overt immunosuppression [1, 6]. A national Swedish cohort study (2008–2018) reported that 3% of CMVR cases occurred in patients with diabetes as the sole risk factor, while hematologic malignancies and hematopoietic stem cell transplantation (HSCT) accounted for 24% and 27% of cases, respectively [4]. Furthermore, a systematic review found underlying malignancies in 29% and diabetes in 6.1% of reported non-HIV CMVR cases, with a significant proportion of patients over 60 years of age [5]. Leukemias, both acute and chronic, contribute through disease-related immunosuppression and intensive chemotherapy, with reported cases in both pediatric and adult populations [7]. 

The clinical features of CMV retinitis in non-HIV immunosuppressed patients often differ from those seen in AIDS-related cases. While the former usually presents with minimal inflammation, CMVR in non-HIV individuals often shows significant vitritis and occlusive vasculitis. When the macula is involved, this can lead to ischemic complications and poorer visual outcomes [5]. 

Diagnosis can be challenging due to its overlap with other retinal diseases, such as diabetic retinopathy or leukemic infiltration. Delays in recognition are common, and definitive diagnosis often requires PCR analysis of ocular fluids. Management involves systemic antivirals and, in some cases, intravitreal therapy, though prolonged immunosuppression and comorbidities complicate treatment [1–4, 8]. 

Importantly, inflammatory complications may persist or emerge after immune recovery. Immune recovery uveitis (IRU), well recognized in HIV-positive individuals, is increasingly observed in non-HIV patients following withdrawal of immunosuppression and CD4 recovery [9]. Recent data suggest that an IRU-like picture may contribute to lasting retinal dysfunction despite virologic control [10]. 

## Case description

A 67-year-old non-smoking diabetic man of Chinese ancestry presented to his local eye department with a three-week history of blurred vision in his right eye. Initial assessment revealed visual acuities of 6/9 in both eyes, along with elevated intraocular pressure of 31 mmHg on the right and 16 mmHg on the left. The patient had a medical history encompassing type 2 diabetes mellitus (DM), hypertension (HT), and hepatitis B seropositive carrier status. Notably, he had previously battled chronic lymphoproliferative disease (B-cell lymphoma) and had achieved remission through a regimen of rituximab, fludarabine, mitoxantrone, and dexamethasone. The last rituximab was given a month before the onset, while fludarabine and mitoxantrone were stopped months before the onset. Negative history of previous eye surgeries.

The clinical examination revealed trace anterior chamber cells in the right eye, without evidence of vitreous activity. Preceding this presentation, the patient had been diagnosed with mild non-proliferative diabetic retinopathy (NPDR) in both eyes. Dilated fundus examination revealed right eye peripheral indolent CMV retinitis (CMVR, around 33% of the retinal surface, affecting zone 1) and intraretinal haemorrhages. The OCT macular scans at presentation revealed an extrafoveal full-thickness necrosis, a swollen and hyperreflective RPE, and a visible Bruch membrane. The choriocapillaris appeared thickened, with a loss of the physiologic dotted pattern. The FFA revealed peripheral occlusive disease and late leakage from retinal veins, secondary to vasculitis. The FAZ was increased in size.

An anterior chamber tap confirmed the presence of cytomegalovirus (CMV) antigens (CMV pp65 in 13 positive cells in 2 × 10^5^ leukocytes). HSV and VZV antigens were negative. We also performed a serum CMV pp65 test, which showed positive serum CMV antibodies. HIV testing was negative.

The positive CMV results prompted the initiation of antiviral therapy per established protocol: intravenous ganciclovir (5 mg/kg q12h for 2–3 weeks, then 5 mg/kg/day) and intravitreal 2 mg ganciclovir (twice a week induction and then weekly maintenance). (*Please see the Flowchart*) After the intravenous ganciclovir regimen, the patient was switched to oral valganciclovir 900 mg BD for three weeks, then 900 mg for maintenance. Three months after presentation, the patient developed cryptococcus-positive pneumonia and needed inpatient antibiotic treatment (intravenous (IV) meropenem followed by oral (PO) voriconazole).

**Fig. 1:**
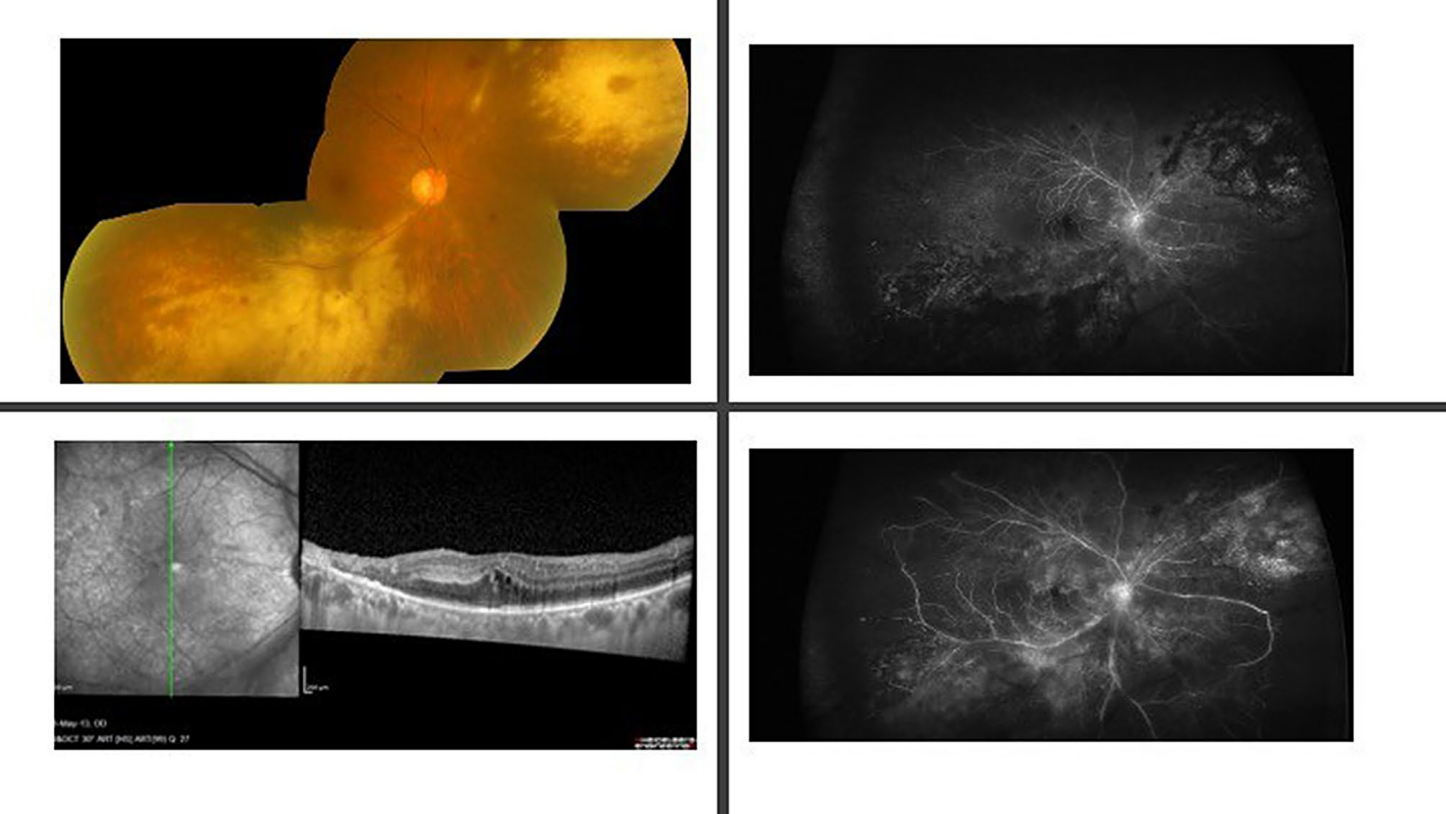
Right eye Colour Fundus, FFA, and OCT images at the time of presentation of presumed immune reconstitution uveitis (IRU). The top left image shows a right eye colour fundus image of the right eye with white necrotic areas affecting posterior pole at presentation. The left bottom image represents the OCT scan of the macula obtained one month after disease onset, demonstrating intraretinal cystoid spaces, signs of resolving retinal necrosis, and a thickened choriocapillaris. The top right image represents the early phase of the right eye fundus fluorescein angiography (FFA), revealing extensive peripheral capillary non-perfusion and an enlarged foveal avascular zone (FAZ). The bottom left image shows a right eye late FFA phase with diffuse vascular leakage, including at the posterior pole, but no evidence of neovascularization – both a month after presentation, after vision dropped to 6/60

Despite commencing antiviral treatment, the patient’s visual acuity in the right eye deteriorated to 6/60, which occurred a month after presentation. Clinical examination revealed no signs of vitreous cells. A repeated fundus fluorescein angiography (FFA) demonstrated an enlarged foveal avascular zone (FAZ) with occlusion of small macular vessels and late-phase macular leakage. (Fig. 1) Additionally, the peripheral retina exhibited extensive areas of large vessel occlusion and capillary non-perfusion. In the late phases, diffuse leakage from larger vessels was observed, without evidence of neovascularization. Consequently, the patient underwent right eye pan-retinal photocoagulation (PRP) laser therapy to address the right eye’s occlusive and non-perfused areas in conjunction with right eye intravitreal anti-VEGF agents (intravitreal aflibercept 2 mg).

Over time, the patient’s disease achieved quiescence, with no evidence of macular edema in the right eye. However, a visually significant posterior subcapsular (PSC) cataract developed, and plans were subsequently made for cataract surgery in the right eye. At quiescence, best-corrected visual acuity was 6/60, limited by macular ischemia confirmed on fluorescein angiography (FFA).

**Flow chart:**
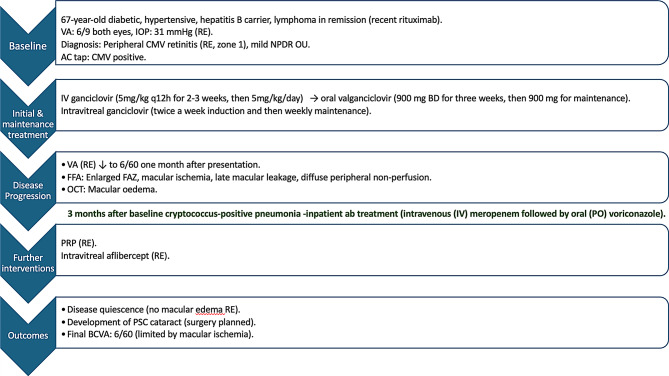
Description: Clinical course and management

## Discussion

This case underscores the complex therapeutic challenges involved in managing CMVR, particularly in HIV-negative patients. The patient exhibited multiple systemic risk factors for non-HIV-related CMV retinitis. Both lymphoproliferative diseases treated with chemotherapy and type 2 diabetes mellitus are independently recognized as predisposing conditions [4–6]. The coexistence of these factors in our patient likely acted synergistically to increase susceptibility to CMVR. Moreover, the presence of mild NPDR suggests that glycemic control was suboptimal, further supporting the contribution of diabetes to the overall risk profile.

Despite initial virologic control with antiviral therapy, the patient developed cystoid macular edema (CME) about one month after presentation. This may have resulted from a combination of diabetic macular edema (DME), or an occlusive vasculopathy–mediated disruption of the inner blood–retinal barrier. Furthermore, CMV has a well-recognized tropism for vascular endothelial cells, and direct viral infection can cause endothelial injury, thrombosis, and vascular occlusion [11]. Such vasculopathy disrupts the inner blood–retinal barrier, promoting ischemia and edema. In this context, the vascular occlusion observed in our case may not solely represent secondary mechanisms but could also reflect the direct pathogenic effects of CMV. Although macular edema is a recognized complication of viral retinitis, it remains relatively uncommon and poses significant management challenges by further worsening visual outcomes.

In our case, the clinical course may raise suspicion of an immune recovery uveitis (IRU)-like response, despite the absence of classical systemic immune reconstitution [9, 10]. This possibility was considered approximately one month after baseline, when the patient’s vision declined and cystoid macular edema (CME) developed. Inflammation following immune recovery can lead to devastating structural damage, particularly in the macula, and has been identified as a primary driver of poor outcomes in these patients [12]. Importantly, the patient had recently discontinued chemotherapy, prompting consideration of whether the withdrawal of immunosuppression triggered an inflammatory rebound [9]. Stopping fludarabine and mitoxantrone, both potent T-cell–depleting agents, may have altered immune dynamics. However, the absence of other overt signs of inflammation, for example, AC or vitreous activity, counts against IR as a contributory cause.

A key clinical dilemma is whether earlier or alternative immunosuppressive therapy could have improved the outcome in our patient. Intravitreal corticosteroids, particularly the dexamethasone implant (Ozurdex), effectively treat uveitis-related macular oedema [13]. However, in HIV-negative patients like ours, diabetic, recently immunosuppressed, and with a recent CMV reactivation, the use of corticosteroids is not straightforward. CMV reactivation following intraocular steroid injection is well documented, and differentiating between active retinitis and IRU-like picture can be diagnostically challenging in real-world settings [14]. Localized intravitreal steroid therapy for the right eye’s cystoid macular edema (CME) could have minimized systemic effects (e.g. risk to the contralateral left eye). Still, the risks of exacerbating the right eye’s CMV retinitis without definitive confirmation of quiescence may have outweighed the benefits of a right eye intravitreal steroid here. Therefore, the decision to defer intravitreal steroids was clinically justified, although it may have contributed to persistent macular inflammation.

For patients unable to tolerate oral corticosteroids or at high risk for reactivation, interferon alpha-2a has been considered as an immunomodulatory alternative [15]. Interferon has shown efficacy in treating cystoid macular edema post-acute retinal necrosis [15]. However, its systemic side effects, limited ocular data, and lack of prospective studies in IRU make it a cautious consideration rather than a mainstream option. Furthermore, interferon alpha-2a has been withdrawn from the market for commercial reasons, limiting its accessibility. In this context, interferon could be reserved for refractory cases or patients intolerant to steroids, but more data are needed to support its routine use.

Pan-retinal photocoagulation (PRP), used to reduce neovascular risk in this patient, is a logical choice in ischemic CMVR with occlusive vasculopathy. However, it does not directly reverse existing macular damage and may exacerbate inflammation without immunosuppression. Its use here was appropriate given the non-perfused periphery, though it did not influence the central visual prognosis.

This case highlights the limited availability of standardized protocols for managing IRU in HIV-negative patients with CMVR. While treatment approaches in AIDS-related CMVR are well established, there is comparatively less consensus for the treatment of IRU-like picture in non-HIV CMVR settings, particularly regarding the timing, route, duration, and choice of immunomodulatory therapy for CMVR-related IRU. Decisions such as when to initiate corticosteroids or how long to maintain concurrent antiviral treatment often depend on individual clinician experience and case-specific factors, reflecting a need for further evidence to guide management in this increasingly recognized patient population.

In conclusion, this patient’s vision loss was most likely attributable to a combination of an IRU-like inflammatory response and severe structural damage from occlusive vasculopathy. These mechanisms, either individually or synergistically, compounded the effects of CMV retinitis and ultimately limited visual recovery despite successful virologic control. Anti-VEGF therapy alone may not sufficiently address any immune-mediated components of the CMVR associated cystoid macular edema (CME). However, in our case, it helped resolve macular edema and maintain vision from further decrease. While intravitreal corticosteroids like Ozurdex may offer benefits, their use in high-risk patients requires caution, especially without confirmed viral quiescence. Interferon alpha-2a presents a biologically plausible alternative but lacks sufficient clinical validation. This case illustrates the pressing need for prospective research, guidelines on inflammation control, and new therapies for CMVR beyond the HIV population. There is considerable effort worldwide towards the introduction of an effective CMV vaccine [16]. A CMV vaccine would hopefully reduce morbidity from CMVR and possibly act as a new treatment for CMVR. Until then, clinicians must rely on careful clinical judgment, close interdisciplinary collaboration, and individualized treatment strategies to balance immune suppression risks against untreated inflammation’s consequences.

## Data Availability

No datasets were generated or analysed during the current study.

## Abbreviations

AIDS: Acquired Immunodeficiency Syndrome
BD: Bis in Die (twice daily)
CME: Cystoid Macular Edema
CMV: Cytomegalovirus
CMVR: Cytomegalovirus Retinitis
CLD: Chronic Lymphoproliferative Disease
DM: Diabetes Mellitus
DME: Diabetic Macular Edema
FFA: Fundus Fluorescein Angiography
FAZ: Foveal Avascular Zone
HAART: Highly Active Antiretroviral Therapy
HIV: Human Immunodeficiency Virus
HT: Hypertension
HSCT: Hematopoietic Stem Cell Transplantation
IOP: Intraocular Pressure
IRU: Immune Recovery Uveitis
IV: Intravenous
NPDR: Non-Proliferative Diabetic Retinopathy
OCT: Optical Coherence Tomography
PO: Per Os (by mouth, orally)
PRP: Pan-Retinal Photocoagulation
PSC: Posterior Subcapsular Cataract
RPE: Retinal Pigment Epithelium
VEGF: Vascular Endothelial Growth Factor
VZV: Varicella-Zoster Virus
